# Unraveling the impact of primary immunodeficiency disorders on the microbiota of dental caries in children through 16S rRNA gene-based metagenomic analysis

**DOI:** 10.55730/1300-0144.5719

**Published:** 2023-07-23

**Authors:** Bushra Lutf Ahmed AL-KEBSI, Gökhan KARS, Hazal ÖZER, Şükrü Nail GÜNER

**Affiliations:** 1 Department of Molecular Biology and Genetics, Faculty of Science, Necmettin Erbakan University, Konya, Turkiye; 2 Department of Pediatric Dentistry, Faculty of Dentistry, Necmettin Erbakan University, Konya, Turkiye; 3 Department of Pediatric Immunology and Allergy, Meram Medical School, Necmettin Erbakan University, Konya, Turkiye

**Keywords:** Children, dental caries, microbiota, 16S rRNA, immunodeficiency

## Abstract

**Background/aim:**

Dental caries is a frequently occurring and multifactorial chronic disease in children resulting from the interaction of cariogenic bacteria and host susceptibility. The aim of this study was to elucidate the impacts of primary immunodeficiency disorders (PIDs) on microbiota of dental caries in children by 16S rRNA gene-based metagenomic analysis.

**Materials and methods:**

Enrolled in this study were 15 children with primary PID with caries (PID group) and 15 healthy children with caries as a control (CG). The DMFT index, saliva flow rate, and buffering capacity of each participant were assessed before the metagenomic analyses were conducted. For taxonomic profiling, the reads were obtained by high-throughput sequencing of the V3–V4 hypervariable region of 16S rRNA.

**Results:**

The DMFT score, saliva flow rate, and buffering capacity of the groups were similar. The flow rate and buffering capacity had no correlation with the number of species with 95% confidence. The metagenomic analysis resulted in the identification of 2440 bacterial species in all of the samples. Among the 50 most prevalent species present at ≥1% relative abundance, *Prevotella melaninogenica* and *Prevotella salivae* were differentially more abundant in the PID group. The PID group and CG showed similar species richness and evenness, but 4 of the 5 samples with the highest Shannon-Weiner and Inverse Simpson indices belonged to the PID group. The Spearman test results for correlation of the species in the PID subgroups showed that *Prevotella oris* had a positively correlated relationship with both *Scardovia wiggsiae* and *Saccharibacteria* genera incertae sedis.

**Conclusion:**

This study provided insight into the caries microbiota of children with immunodeficiency diseases. Differentially abundant species, novel bacterial associations, and unique bacterial species were disclosed in the PID samples, indicating the role of the immune system in altering the caries microbiota. The prominent bacterial species and associations in the PID group should be suspected in regard to their link with present or future diseases.

## 1. Introduction

Dental caries is a frequently occurring chronic disease in children, irrespective of their socioeconomic status. It is a multifactorial disease resulting from the interaction of a cariogenic diet, cariogenic bacteria, and host susceptibility. *Streptococcus mutans* is regarded as the primary pathogen for caries but other species of *Streptococcus*, *Lactobacillus*, *Actinomyces*, and *Veillonella* have also been associated with the disease [1]. While determining the caries risk, the saliva flow rate and buffering capacity, which are important factors, should be evaluated together. The saliva flow rate affects the content of the saliva [2]. Among the reasons for the lack or decrease of secretion are immune deficiencies, diabetes, sialolithiasis, sarcoidosis, Sjögren’s syndrome, surgical removal of the salivary glands, radiotherapy, and the use of atropine-like drugs [3–5]. One of the essential functions of saliva in preventing dental caries is to neutralize and buffer the organic acids formed in the mouth. pH is a variable that should be considered in regard to caries activity [6]. Individuals with a high buffering capacity are resistant to caries formation [7]. The pH of saliva is slightly acidic when first secreted. When saliva is stimulated, its buffering capacity increases [5].

Isolation and culturing techniques were designed to isolate few known bacteria compared to a metagenomic approach, which provides a broad-spectrum analysis of microbiota. Next-generation sequence technologies have enabled the high-throughput analysis of microorganisms in different niches of the oral cavity with no need for bacterial culturing. It was revealed with metagenomic analyses that the oral cavity may accommodate more than 700 species, many of which are responsible for periodontal disease, biofilm formation, and tooth decay [8]. In addition to oral diseases, oral pathogens have been strikingly linked to several systemic diseases, such as bacterial pneumonia, infective endocarditis, rheumatoid arthritis, colon cancer, and inflammatory bowel disease [8].

Primary immunodeficiency disorders (PIDs) refer to disorders related to the immune system, whether functionally or developmentally. Most PID patients have a high risk of susceptibility to infection and an early diagnosis and treatment are critical to prevent morbidity [9]. The immune system has an influence on host-microbe homeostasis [10] and bacterial behavior in the environment and hence, it has roles in initiating and developing dental caries [11]. Oral microbiota is second, after gut microbiota, and contains the largest number and diverse bacterial species, which make it an important part of the human microbiota. The oral cavity is physically connected to significant parts of the body, such as the respiratory and gastrointestinal tracts and hence, it has potential impacts on their microbial composition. Understanding oral microbiota comprehensively in both healthy and diseased individuals is advantageous to understanding any alterations that may impact health. Bacterial taxonomic composition analysis can be an indicator of oral health status, and prominent bacterial species and associations can be used as potential biomarkers of oral disease, especially in PID patients [12]. Recently, many studies have been conducted to unveil the link between gut microbiota and illnesses such as cancer and autoimmune diseases [8]. Similarly, there is a need to disclose the impact of PIDs on the caries microbiota of patients with these diseases so that preventive and remedial approaches can be implemented.

The purpose of this study was to elucidate the impact of PIDs on the microbiota of dental caries in children suffering from these diseases via a 16S rRNA gene-based metagenomic approach and compare the results to those obtained from the healthy group.

## 2. Materials and methods

### 2.1. Participants and clinical examinations

Enrolled in this study were 15 patients (n = 15, 7 females and 8 males, mean age: 6.5 ± 3.3 years) with PIDs (PID group) and 15 patients (n = 15, 7 females and 8 males, mean age: 6.9 ± 1.7 years) as the control group (CG) who attended the Pedodontics Clinic of Necmettin Erbakan University, Faculty of Dentistry, Konya, Türkiye. Clinical examinations and the collection of saliva and decayed teeth samples were done in a period of 2 months at the aforementioned clinic for the 30 patients. The patients with PIDs were further divided into subgroups as the antibody deficiency (PID1, PID2, PID8, PID9, PID12, PID14, and PID15; n = 7), immune dysregulation (PID4, PID10, and PID11; n = 3), severe combined immunodeficiency (SCID) (PID3 and PID6; n = 2), and SCID with stem cell transplantation (PID5, PID7, and PID13; n = 3) groups.

When assessing caries risk, it is crucial to evaluate both the saliva flow rate and buffering capacity together to ensure an accurate evaluation. These factors are essential and must be considered in tandem [13]. Saliva samples were taken from patients after at least 1 h of not consuming any food or liquids, or brushing their teeth. To stimulate saliva production, each patient was given a paraffin pellet to chew on, and then saliva was collected from the floor of the mouth into sterile tubes. The saliva flow rate was then calculated as the milliliters of saliva that accumulated in 1 min (mL/min). The buffering capacities of the saliva samples were measured with disposable test strips according to the manufacturer’s instructions (CRT buffer, Ivoclar Vivadent, Schaan, Liechtenstein). For this, the test strips were moistened with saliva and the saliva buffering capacity was evaluated after 5 min by visual assessment as high (blue), medium (green), or low (yellow). The decayed, missing, and filled teeth (DMFT) values were evaluated as a result of intraoral and radiographic examinations.

### 2.2. Dental caries sample collection and DNA extraction

The dental caries samples were collected by a trained pediatric dentist with clinical and research experience at the Pedodontics Clinic of Necmettin Erbakan University, Faculty of Dentistry, Konya, Türkiye. Teeth with the indication for tooth extraction were extracted with the application of topical anesthesia (Xylocaine, Astra, Södertalje, Sweden) and then local anesthesia (Ultracain, Aventis Pharma, İstanbul, Türkiye). They were preserved in a sterile tube at 4 °C for further DNA extraction within 1 week. The samples were washed with 70% ethanol and the dental caries were ground into powder using a mortar and pestle. Afterwards, DNA extraction was done using a kit (ZymoBIOMICS DNA Miniprep kit, Tustin, USA) following the manufacturer’s instructions. The quantification and purity of the DNA were assessed using a NanoDrop spectrophotometer (NanoDrop, Thermo Scientific, Wilmington, DE, USA). Aliquots of purified DNA samples (100 ng DNA) were shipped to an external company (GenEra, İstanbul, Türkiye) for metagenomics analysis.

### 2.3. 16S rRNA gene-based metagenomic analysis

The gene-specific sequences used in this protocol targeted the V3 and V4 regions of the 16S gene, stated as the most promising region in terms of coverage [14]. The sequences of the primer pairs used for the amplification of these regions were as follows: 16S amplicon polymerase chain reaction (PCR) forward primer (Illumina_16S_341F): 5′ TCGTCGGCAGCGTCAGATGTGTATAAGAGACAGCCTACGGGNGGCWGCAG and 16S amplicon PCR reverse primer (Illumina_16S_805R): 5′ GTCTCGTGGGCTCGGAGATGTGTATAAGAGACAGGACTACHVGGGTATCTAATCC. Illumina adapter nucleotide sequences flanking the gene-specific primers were underlined. Next, 2-step PCR was performed for the library preparation. In these procedures, 25 cycles of PCR were performed separately for each sample using KAPA HiFi HotStart ReadyMix (Roche Diagnostics, Basel, Basel-Stadt, Switzerland). The first PCR step consisted of 3 min at 95 °C, and then 95 °C for 30 s + 55 °C for 30 s + 72 °C for 30 s, for 25 cycles. And finally, a single cycle was applied at 72 °C for 5 min. In the following PCR, the Nextera XT index primer 1 and Nextera XT index primer 2 sets (Illumina) were used to add the Illumina index and adapter sequences. This PCR sequence consisted of 3 min at 95 °C, and then 95 °C for 30 s + 55 °C for 30 s + 72 °C for 30 s, for 8 cycles. And finally, a single cycle was applied at 72 °C for 5 min. After each PCR, purification was performed with the magnetic beads (AMPure XP; Beckman Coulter Life Sciences, Indianapolis, IN, USA). At this stage, the PCR products alone did not have any meaning; therefore, this library was then sequenced with the Illumina iSeq 100 next-generation sequencing platform (Illumina Inc., San Diego, CA, USA) through paired-end (2 × 150 bp) reading using the iSeq 100 i1 Reagent kit, following the manufacturer’s instructions (Gen-Era Diagnostics, İstanbul, Türkiye). These sequences or reads were aligned to target microorganisms using the bioinformatics tools explained in the following section.

### 2.4. Statistical and bioinformatics analyses

After sequencing, FastQC 0.11.9 (Babraham Bioinformatics Group, Babraham Institute, Cambridge, UK) was used for the quality control processes. According to the QC results, the amount of data, read quality, guanine-cytosine (GC) distributions, *k-*mer distributions, and possible adapter contaminations were examined for each sample. After that, reads with poor read quality (Phred score <Q20, window range of 30 bp) were excluded from the data. Furthermore, low-quality base reads, possible adapter contaminants, and chimeric sequences at the read tips were trimmed based on the Genomes Online Database (GOLD) using the Trimmomatic tool (version 0.40, usadellab.org, Germany) developed by Bolger et al. [15].

For taxonomic profiling, the reads were aligned to target organisms based on the Greengenes Database using the Ribosomal Database Project (RDP) classifier [16]. After alignment, the operational taxonomic units in each sample were determined for the microbial community structure and composition. Bioinformatics analyses such as analysis of the alpha and beta diversity, data reporting, statistical analysis, and data visualization were carried out using R vegan 2.5–6 (https://CRAN.R-project.org/package=vegan). The differences in the relative abundances of taxa between the study groups were explored using the t test, Kruskal-Wallis test, and Mann-Whitney U test, and in addition, the Spearman test was used for correlation to evaluate the relationships between species. An adjusted p < 0.05 was considered significant. IBM SPSS Statistics for Windows 29.0 (IBM Corp., Armonk, NY, USA) was used for detailed statistical analyses.

## 3. Results

### 3.1. Clinical examinations of the participants

Laboratory tests findings of the 15 PID patients with caries and the 15 healthy children in the CG are shown in the Table. The mean DMFT values of the groups were relatively similar with no significant difference (p > 0.05). Moreover, the saliva test results showed no differences between the groups regarding the saliva buffering capacity (medium) and flow rate (PID = 0.9 ± 0.4 mL/min and CG = 1 ± 0.4 mL/min). The mean number of species for the PID group and GC were 410 ± 171.3 and 395 ± 165, respectively (Table). Furthermore, any correlation between the number of species and saliva flow rate and buffering capacity was examined and no significant correlation was predicted, neither between the buffering capacity and bacterial species (R^2^ = 0.06, F(1.28) = 1.78, p = 0.19) nor between the flow rate and number of bacterial species (R^2^ = 0.04, F(1.28) = 1.19, p *=* 0.3).

### 3.2. Overall microbial profile

**Figure 1:**
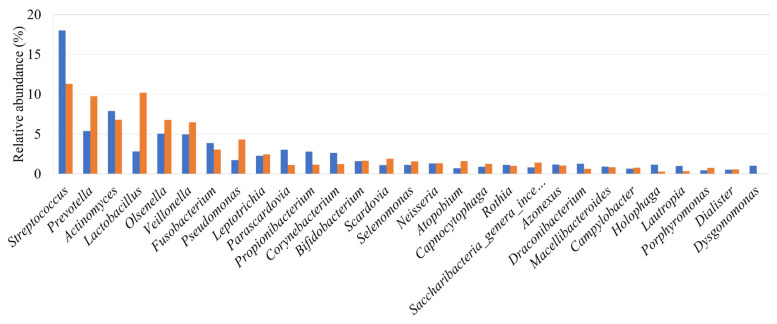
Relative abundances of the major genera identified in the CG ( 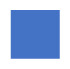) and PID group ( 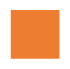).

**Figure 2:**
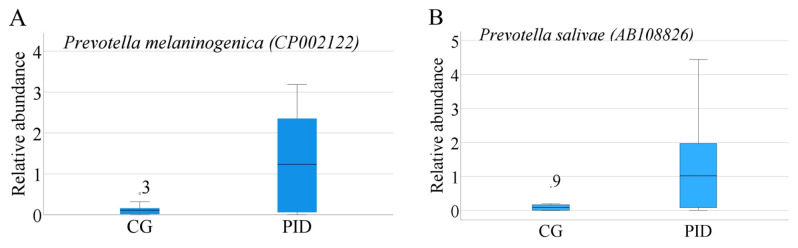
Differentially abundant bacterial species [*P. melaninogenica* (a) and *P. salivae* (b)] between the CG and PID group.

**Figure 3:**
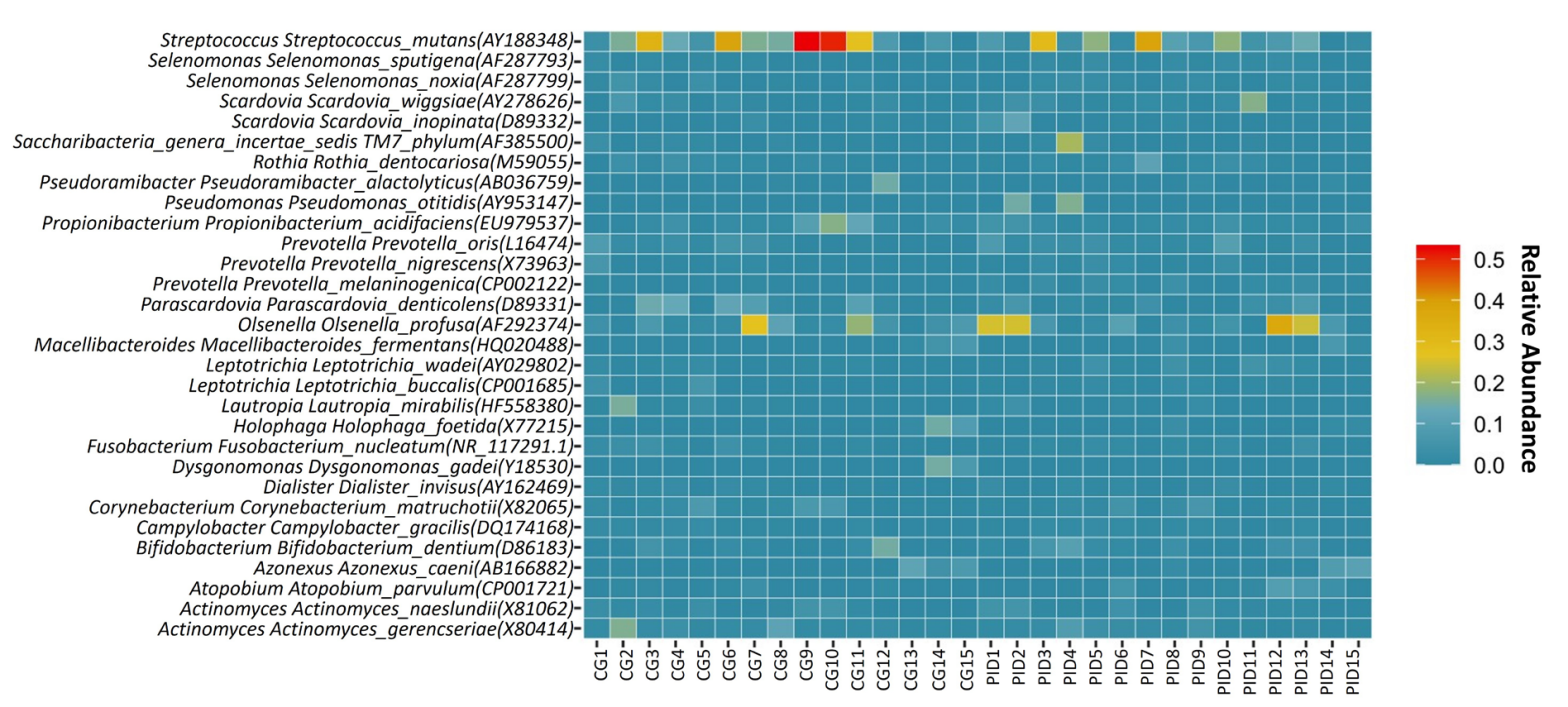
Heat map of the relative abundances for the 30 most abundant bacterial species in each study group.

Reads that passed quality control were assigned to the taxonomic unit with the highest similarity at various taxonomic levels. The analysis of all of the samples resulted in the identification of 2440 bacterial species belonging to 1327 genera, 303 families, 123 orders, 82 classes, and 46 phyla. Figure 1 depicts the relative abundances of the major genera identified in all of the samples. Among the most relatively abundant genera, those present at ≥1% relative abundance in both groups were as follows: *Streptococcus* (18.03% and 11.32%), *Prevotella* (5.39% and 9.77%), *Actinomyces* (7.92% and 6.82%), *Lactobacillus* (2.82% and 10.20%), *Olsenella* (5.04% and 6.79%), *Veillonella* (4.97% and 6.49%), *Fusobacterium* (3.88% and 3.06%), *Pseudomonas* (1.72% and 4.32%), *Leptotrichia* (2.28% and 2.46), *Parascardovia* (3.03% and 1.12%), *Propionibacterium* (2.79% and 1.17%) and *Corynebacterium* (2.65% and 1.24%), *Bifidobacterium* (1.60% and 1.65%), *Scardovia* (1.11% and 1.91%), *Selenomonas* (1.13% and 1.57%), *Neisseria* (1.32% and 1.33%), *Rothia* (1.13% and 1.02%), and *Azonexus* (1.19% and 106%) for the CG and PID group, respectively. The number of bacterial species/strains per sample ranged from 235–820. Differentially abundant species between the PID and CG groups were determined by applying the Kruskal-Wallis test among the 50 most abundant species (Figure 2). No significant differences, except for *Prevotella melaninogenica CP002122* (p = 0.002) (Figure 2a) and *Prevotella salivae AB108826* (p = 0.004) (Figure 2b), which were found to be associated with the PID group, were detected between the groups. The heat map of the relative abundances for the 30 most abundant bacterial species in each study group is illustrated in Figure 3. Among most abundant species, those present at ≥1% relative abundance in both groups were as follows: *S. mutans* (AY188348) (19.13% and 11.1%), *Olsenella profuse* (AF292374) (6.12% and 9.63%), *Parascardovia denticolens* (D89331) (4.14% and 1.84%), *Propionibacterium acidifaciens* (EU979537) (3.64% and 1.84%), *Actinomyces gerencseriae* (X80414) (2.97% and 1.96%), *Prevotella oris* (L16474) (1.79% and 2.41%) *Bifidobacterium dentium* (D86183) (1.87% and 2.05%), *Corynebacterium matruchotii* (X82065) (1.93% and 1.26%), *Actinomyces naeslundii* (X81062) (1.48% and 1.58%), *S wiggsiae* (AY278626) (1.13% and 2.25%), *Azonexus caeni* (AB166882) (1.59% and 1.42%), and *Rothia dentocariosa* (M59055) (1.04% and 1.56%) for the CG and PID group, respectively.

### 3.3. Bacterial diversity of the tooth caries

**Figure 4:**
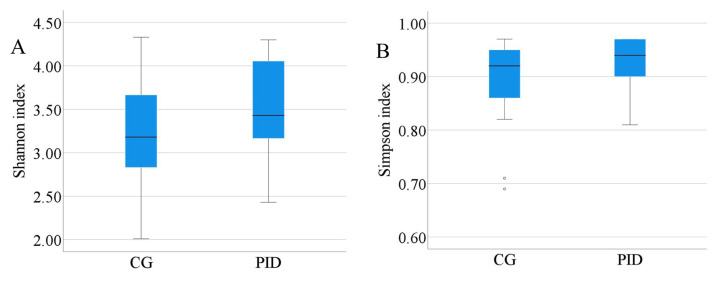
Comparison of the observed Shannon-Weiner (A) and Simpson (B) indices between the CG and PID group.

**Figure 5:**
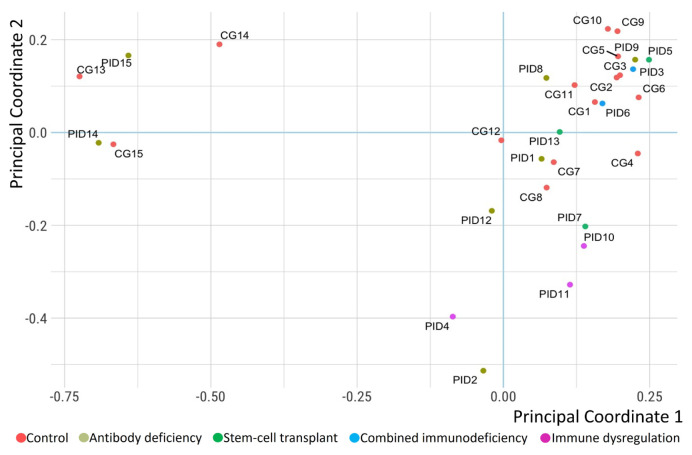
Principal coordinate analysis (PCoA) of the normalized relative microbial diversity of the study groups based on the microbial profile at the genus level.

The Shannon-Weiner and Simpson indices were calculated for each sample to investigate the alpha diversity of the caries microbiota. Then, the mean diversity indices of the CG and PID group were compared using the t test (Figure 4). As a result of the comparison, no statistically significant difference was found regarding the Shannon-Weiner index (PID group (M = 3.51; SD = 0.58) and CG (M = 3.20; SD = 0.68), t (28) = −1.36, p = 0.19) (Figure 4A) or Simpson index (PID group (M = 0.92; SD = 0.05) and CG (M = 0.89; SD = 0.09), t (28) = −1.30, p = 0.21) (Figure 4B). Moreover, the Shannon-Weiner and Simpson indices for each PID subgroup had no significant differences, as determined using the Kruskal-Wallis test (p > 0.05). For the beta diversity measurement, principal coordinate analysis (PCoA) based on the microbial profile at the genus level was employed to examine any variations in caries microbiota among the samples of the PID group and CG. As shown in Figure 5, samples that exhibited similar microbial distribution formed clusters.

### 3.4. Bacterial composition in the PID subgroups and correlation of the abundant species

**Figure 6:**
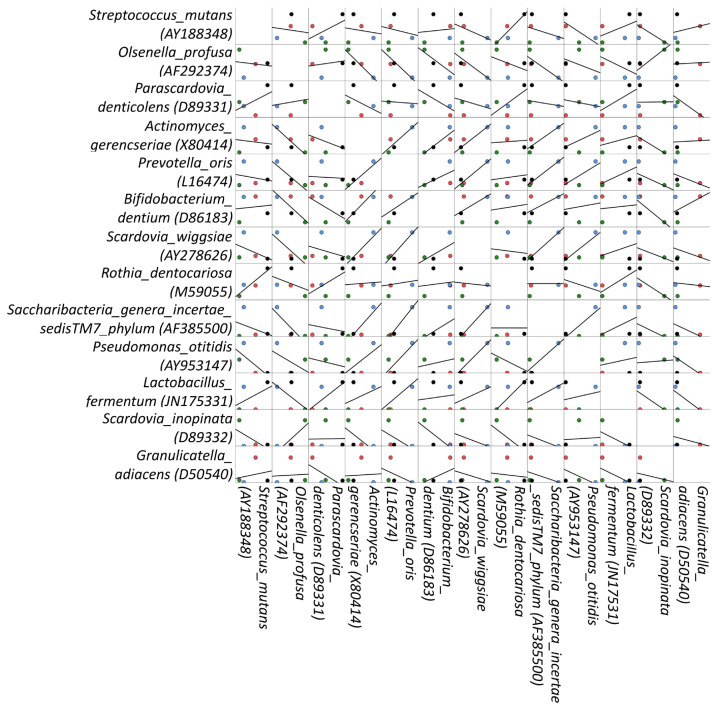
Spearman test results showing the correlation of the species in combined immunodeficiency ( 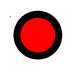), antibody deficiency ( 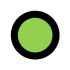), immune dysregulation ( 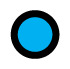), and stem cell transplantation ( 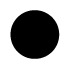).

In this part of the study, the bacterial species in each of the PID subgroups were examined and any correlation of the species were disclosed. Species that had >3% abundance in the PID subgroups were *Granulicatella_adiacens* (D50540), *Scardovia_inopinata* (D89332), *Lactobacillus_fermentum* (JN175331), *Pseudomonas_otitidis* (AY953147), *Saccharibacteria_genera_incertae_sedis TM7_phylum* (AF385500), *Rothia_dentocariosa* (M59055), *Scardovia_wiggsiae* (AY278626), *Bifidobacterium_dentium* (D86183, *Prevotella_oris* (L16474), *Actinomyces_gerencseriae* (X80414), *Parascardovia_denticolens* (D89331), *Olsenella_profusa* (AF292374), and *Streptococcus_mutans* (AY188348). *Lactobacillus fermentum* (JN175331) and *Macellibacteroides fermentans* (HQ020488) were the 2 noticeable bacterial species in the tooth caries of the patients with antibody deficiency. In addition, *P. denticolens* (D89331), *S. wiggsiae* (AY278626), *Atopobium parvulum* (CP001721), *Prevotella nigrescens* (X73963), and *Prevotella histicola* (EU126661) were the marked species in the tooth caries of the patients with combined immunodeficiencies but received stem cell transplantation. The Spearman test was applied to reveal any correlation of the species that had >3% abundance in the PID subgroups. The analysis depicted that *P. oris* had a positively correlated relationship with both *S. wiggsiae* and *Saccharibacteria* genera incertae sedis (p < 0.05). In addition, the positive correlation between *S. wiggsiae* and *Saccharibacteria* genera incertae sedis alone was more significant (p < 0.01) (Figure 6).

## 4. Discussion

To our knowledge, this is the first detailed report on elucidating the impact of immune deficiency diseases on bacterial microbiota of dental caries. In addition to the clinical examination of the participants, the bacterial composition, diversity, correlation, and coprevalence of the abundant species in both the CG and the PID subgroups were investigated from different perspectives. Notable results were discussed by considering the potential clinical impacts of the prominent bacterial species. The clinical features (saliva buffering capacity, saliva flow rate, and DMFT) of the PID group and CG were similar with no significant difference. Furthermore, the number of bacterial species in the groups was also quite similar. It was observed that aside from the immunodeficiency state, the eating habits and general self-care habits, socioeconomic status, general health status, and accompanying diseases might all contribute to these clinical consequences; and thereby, such homologous outcomes may emerge. In the initial comparison of the caries microbiota of the immunodeficient patients and the CG, *S. mutans* (AY188348), a major etiologic agent in dental caries, was observed as the most prevalent species among all, and the prevalences of *P. melaninogenica* and *P. salivae* were higher in the PID group. *P. melaninogenica* is one of the clinically important anaerobic gram-negative bacilli (AGNB). It is one of the species present in oral microbiota and is known for lung-related diseases in addition to abscess formation [17]. In one study, *P. melaninogenica* was found to be predominant in caries-active children compared to those who did not have caries [18]. It was also reported that the elevated abundance of *Prevotella* activated toll-like receptors that triggered the immune response by antigen-presenting cells that produced T helper type 17 (Th17), including interleukin 23 (IL-23) and IL-1 [19]. In addition, *Prevotella* was said to stimulate epithelial cells to produce IL-8 and IL-6, which induce neutrophils [10, 19]. In regard to the current study, it can be stated that the cells of the immunocompromised patients may not have properly responded to *P. melaninogenica* and therefore, may not have activated an immune response, which, in turn, may have resulted in a higher prevalence of the bacterium.

Kıykım et al. [20] conducted a similar study where the children with PID and healthy children were all from Türkiye. In their study, the oral microbiota of selective immunoglobulin A (IgA)-deficient patients and X-linked agammaglobulinemia (XLA) patients were compared to those of healthy group. Upon oral cavity examination, there was no significant difference in the frequency of dental caries, the presence and history of intraoral lesions, or plaque and gingival index values among the children participating in the study. Moreover, there was no significant difference in terms of the *S. mutans*, *Lactobacillus*, or yeast levels among the patients. Only the *Lactobacillus* level was higher in the XLA patients than in the selective IgA-deficient patients. The authors remarked that compromised elements of the immune system, such as secretory IgA deprivation, might have been compensated by other salivary and mucosal defense factors in the host innate immune system, so that balanced oral microbiota was achieved without a distinct difference between the groups. In another study from Türkiye, the oral health status of children with and without autism spectrum disorder (ASD) was compared and no statistically significant difference was found between the ASD and non-ASD groups in terms of the *S. mutans* and *Lactobacilli* loads in the saliva [21]. These aforementioned studies showed more or less similar microbial profiles in the subjects despite the presence of small differences.

In the context of alpha diversity measured by the Shannon-Weiner and Simpson indices, similar species richness and species evenness were observed in the PID group and CG, but it was also noted that 4 of the 5 samples with the highest Shannon-Weiner and Simpson indices belonged to the PID group. This finding might be interpreted as showing that a compromised host immune system could lead to greater bacterial diversity. Similar arguments were also valid for the beta diversity measurements. While the PCoA did not clearly show clustering of the PID subgroups as a whole (Figure 5), it was observed that 2 or more individual samples did cluster together. For instance, PID2 and PID4, and PID7, PID10, and PID11 were positioned close to each other in the PCoA.

Herein, certain prominent bacterial species in the PID subgroups were further examined regarding their roles in caries genesis and special features. For instance, *M. fermentans* (HQ020488) is a gram-positive obligately anaerobic bacterium [22], which has been found often in patients with antibody deficiency. *M. fermentans* was reported to be found in granulation tissue samples of patients with chronic periodontitis [23]. To our knowledge, the present study showed its prevalence in the caries of children for the first time. Patients with combined immunodeficiencies but who received stem cell transplantation demonstrated considerably high abundances in *P. denticolens* (D89331) and *S. wiggsiae* (AY278626). *P. denticolens*, which was previously named *Bifidobacterium denticolens*, is a gram-positive, nonmotile anaerobic bacterium [24, 25]. It is a member of *Bifidobacteriaceae*, whose representatives were reported to be isolated from caries [25, 26], gingival crevices [27], and saliva [28]. Its prevalence was reported to be similar in both plaque and caries [26]. In a study done by Reyman et al. [29], the role of microbial community networks throughout parts of the body in susceptibility to respiratory tract infections (RTIs) was disclosed and cariogenic *P. denticolens* was one of the key species in the most susceptible RTI networks. In the current study, the high prevalence of *P. denticolens* in the patients with stem cell transplantation may also be considered as an indication and it may be recommended that these patients are examined to evaluate their predisposition to RTIs. *S. wiggsiae*, which was also a highly abundant species in the patients with stem cell transplantation is a gram-positive, anaerobic, nonmotile bacillus isolated from the oral cavity of humans [30]. Similar to *P. denticolens*, *S. wiggsiae* is a member of *Bifidobacteriaceae*. It is known for reducing the pH of the environment by utilizing glucose and producing acetic acid, which contributes to the demineralization of teeth [31]. It was strongly associated with severe early childhood caries in children, and it was proposed as a candidate caries pathogen for early childhood caries [1]. However, there has been no evidence thus far in regard to its association with any diseases other than plaque and caries formation. *A. parvulum* (CP001721), *P. nigrescens* (X73963), and *P. histicola* (EU126661) were also encountered often in the caries of the patients with stem cell transplantation. *A. parvulum* is a gram-positive, nonmotile, and obligate anaerobic bacterium that has been associated with halitosis and human oral infections [32]. The prevalence of *A. parvulum* in humans has commonly been linked to caries [32, 33]. However, in one study, it was interestingly assigned as the key species in the hub of H_2_S producers in the human intestine, and its relative abundance was positively correlated with the severity of Crohn’s disease [34]. To our knowledge, this is the first report showing that *A. parvulum* was quite abundant in patients with stem cell transplantation. *P. nigrescens* is a gram-negative and obligately anaerobic bacterium [35]. It has been associated with healthy oral cavities [36], but it has also been found in chronic endodontic infections [37] and periodontal disease [38]. *P. nigrescens* was stated to have crucial roles in subgingival plaque together with *P. intermedia*. *P. histicola* isolated from the human oral cavity is a gram-negative, obligately anaerobic, and nonmotile bacterium [39]. The abundance of *P. histicola* was reported to be much higher in the supragingival plaque samples of children with caries than in those without [40]. Similar findings were also discovered in a study where this bacterium was more prevalent in the saliva of children with caries than in that of children without [41]. *Prevotella* is a genus whose members can be found in oral and gut microbiota [40–42]. In a study done by Mangalam et al. [42], a gut-derived *P. histicola* was used to suppress multiple sclerosis (MS) in a preclinical animal model of MS. Together with these results, it is seen that *P. histicola* may have a dual role, causing diseases in the oral cavity, while also acting as a therapeutic agent for diseases in other parts of the body.

The prevalence of certain bacterial species, on the other hand, varied irrespectively from the immune state of the host. For instance, *A. naeslundii* (X81062), *Lautropia mirabiils* (HF558380), *Prophyromonas endodontalis* (AY253728), and *Streptococcus mutans* (AY188348) were quite common in the tooth caries of the CG. Vielkind et al. [43] investigated the prevalence of *Actinomyces spp*. in patients with chronic periodontitis and found *A. naeslundii* as one of the most prevalent species in both the patients and healthy subjects. Similarly, *L. mirabilis* was notably associated with healthy oral microbiota [18, 44, 45], such that it was one of the most prevalent species in the supragingival plaque of children without caries [18]. *Porphyromonas endodontalis* is a black-pigmented, biofilm-forming, anaerobic bacterium associated with endodontic and periodontal lesions [46]. These strict anaerobic bacteria with minimal nutrient requirements are located within the root canal where they shelter themselves from the host cellular and humoral immunity [46, 47]. In addition to this, the ability of these bacteria to form biofilms also helps them to evade the host organism’s immune system. Therefore, it can be inferred from the above findings that the presence and richness of bacterial species may not be solely associated with the immunological status of the host. A single type of defect in the immune system may be compensated by other elements of the immune defense mechanism, as well.

Correlation analysis is one of the engrossing parts of microbiota studies, as an exceptional association between species may be encountered. Herein, the Spearman test was applied to reveal any correlation of species that had >3% abundance in the PID subgroups. The analysis depicted that *P. oris* had a positively correlated relationship with both *S. wiggsiae* and *Saccharibacteria* genera incertae sedis (p < 0.05). In addition, the positive correlation between *S. wiggsiae* and *Saccharibacteria* genera incertae sedis alone was more significant (p < 0.01) (Figure 6). In another study, a very tiny member of Candidate Phyla Radiation phylum *Saccharibacteria*, known as TM7x, was found to have a parasitic association with its host, *Actinomyces odontolyticus* strain XH001, in the human oral cavity and stably existed as a coculture [48]. Based on these results, it can be remarked that members of *Saccharibacteria* seem to have a tendency to form close associations with other species in the oral cavity and the impact of this association on patients with immunodeficiency requires further investigation.

Recently, there has been an increasing amount of research to reveal any linkage between diseases and presence of unique bacterial species in various parts of human body [8, 49, 50]. For instance, *Subdoligranulum didolesgii* was suspected of developing rheumatoid arthritis, an autoimmune and inflammatory disease [49]. In another study, associations between oral microbiota and several types of cancers, such as head, neck, and prostate, were established [50]. All of these findings support the hypothesis that presence of unique bacterial species or any microbial dysbiosis in any organs should be considered carefully for the prevention of diseases. The present study also contributes to these studies by unveiling unique bacterial species and novel bacterial associations in the PID group.

Regarding the limitations of the study, the present work was done with a relatively small population size in a city of Türkiye by a locally supported project. The results are challenging, as unique bacterial species and novel bacterial associations were disclosed but further experimental studies could be repeated with much greater sample sizes through international collaborations so that a more generalized outcomes could be obtained. Second, in addition to bacterial microbiota, eukaryotic microbiota of the caries may further be studied to have additional outcomes.

In conclusion, bacterial microbiota of the tooth caries of both the PID group and CG were revealed through 16S-based metagenomic analyses in addition to the investigation of the clinical features of the patients, such as the saliva buffering capacity, saliva flow rate, and DMFT. In addition to several similarities in the diversity measurements and results of the clinical examinations, unique bacterial species were also identified. Further investigation of the PID subgroups brought to light various cariogenic bacteria, some of which were observed in caries for the first time in the literature. Moreover, novel bacterial associations were revealed in the caries microbiota of primary immunodeficient children upon correlation analysis. In future studies, these findings could be utilized to anticipate any onset of diseases or disease-bacterial associations in PID patients.

## Figures and Tables

**Table:** Laboratory test findings of the study participants.

	Mean ± SD		
	CG	PID group	p-value
Saliva buffer capacity	Medium	Medium	0.5
DMFT	9.6 ± 3.6	8.3 ± 2.3	0.3
Saliva flow rate	1 ± 0.4	0.9 ± 0.4	0.4
Species number	410 ± 171.3	395 ± 165	0.8

DMFT: decayed, missing, and filled teeth.
